# Accountability and transparency are vital in a pandemic response

**DOI:** 10.1002/jgf2.401

**Published:** 2020-11-11

**Authors:** Kazuki Shimizu, Elias Mossialos

**Affiliations:** ^1^ Department of Health Policy London School of Economics and Political Science London UK; ^2^ Faculty of Public Health and Policy London School of Hygiene and Tropical Medicine London UK; ^3^ Graduate School of Medicine Hokkaido University Sapporo Japan

**Keywords:** data sharing, governance, health communication, health emergency, health security, infectious disease epidemiology

To the Editor,

In the early phase of the coronavirus disease 2019 (COVID‐19) pandemic, an operation of the Diamond Princess cruise ship in Japan in February‐March 2020 became a critical humanitarian topic in global health. We read the recent editorial by Tokuda et al with great interest that vividly depicted an obscure command and control system on the frontline and an inadequate infection control.^1^ These challenges, combined with the infectiousness of COVID‐19 in the asymptomatic phase, made it demanding to contain the virus immediately. Here, we discuss additional major challenges untouched.

First, passengers and crew were not equally treated during the quarantine period. Epidemiological studies supported this argument: The reproduction number among passengers promptly declined to below one on February 5, 2020, when the quarantine operation started; however, the transmission spread among crew members,^2^ as they had to maintain their services.

Second, transparency on the extent and handling of the infection was insufficient. It took 2 weeks for the National Institute of Infectious Disease to publish the first epidemiological report written in English, followed by the press conference by epidemiologists on February 21, 2020. Moreover, as many key epidemiological findings have only been published in Japanese, it became difficult for global researchers, media, and citizens to access the latest scientific insights and incorporate them into their responses. This example of insufficient transparency and data sharing might have triggered the harsh criticism toward Japan's handling from the foreign press.

Third, minimal accountability with obscure governance of health emergency planning had become evident. Who made the decision of each countermeasure and on what purposes has remained unclear even as of today.^1^ The decision‐making process was dominated by ministers and bureaucrats who were amateurs in infection control, and opinions from experts were sometimes rejected.^1^

Despite these challenges, Japan defended that “the operation was improved timely and appropriately” in the Diamond Princess report,^3^ without presenting implications for future outbreaks inside the cruise ship. This report is currently only published in Japanese, and key lessons learned from the unprecedented operation are self‐complacent. A lack of accountability and transparency is still obvious in Japan's domestic response to COVID‐19.^4^

The outcome of the Diamond Princess operation suggests that raising preparedness for health emergencies in enclosed spaces is vital. Several COVID‐19 outbreaks have been already reported in cruise ships, and preventing further outbreaks and improving its management are critical. International solidarity is pivotal in the COVID‐19 response,^5^ and Japan, a promoter of global health security, must take initiative to promptly share scientific insights of COVID‐19 in enclosed spaces; scrutinize the previous unprecedented operation; openly summarize challenges in infection control, health communication, governance, international law, or other realms; and act on lessons learned. Launching an independent panel and conducting an external evaluation will help achieve this process. This will not only strengthen Japan's preparedness and response toward emerging infectious diseases, but also globally improve future responses toward health emergencies in cruise ships, mitigate their impact, and protect the health of passengers and crews.

## CONFLICT OF INTEREST

The authors have stated explicitly that there are no conflicts of interest in connection with this article.
